# Therapeutics and the Lesser Circulation

**Published:** 1895-01-26

**Authors:** Benjamin Ward Richardson


### THERAPEUTICS AND THE LESSER CIRCULATION.

By SIR BENJAMIN WARD RICHARDSON, M.D., F.R.S.

(*Continued from page* 217.)

III.

AT the close of the last part of this subject, I arrived at the vital question whether there is any immediate effect in the administration of medicines upon the vascular, or nervous, system of the lungs themselves; or whether the varying conditions produced in the lungs are dependent purely on the influence of the heart acting on simple passive structures, and indicate more cardiac pressure, or cardiac feebleness.

I do not think that in the whole range of therapeutics there is a question more elementary, more important, or more unsettled than this. It is the most common of events that, in the treatment of diseases of all kinds, death commences in the lesser circulation. Independent life commences in the lesser circulation. Death commences there, and is usually determined from that point. There are, of course, other centres in which the phenomena of death commence, as when there is a deposit of fibrine on the right or left side of the heart, or when there is injury to the spinal cord in its upper part, or the medulla, or brain; or when there is rupture of a large blood-vessel; but, putting aside these comparatively rare forms of death, and taking the whole run of cases of death, how general it is that we find the lungs the centre of the impending change. They are congested in their vascular structure; they are emphysematous; they are surrounded with effused fluid, or their bronchial surfaces are filled with secretion so that the body dies—drowned, as it were, in its own fluid, *hydrops bronchialis*. In the aged and enfeebled death appears to take place—I had almost said naturally—from the lungs, so that life seems to be extinguished by a kind of natural anæsthesia. The muscular inspiratory power is low, the elastic structure of the lungs is enfeebled, and slight variations of external heat and cold suffice to produce the most determinate changes. Cold, especially, becomes the grand obstructor of vital respiration, enfeebling muscular energy, enfeebling elastic resilience, favouring condensation of fluid on the bronchial surface, interfering with oxidation, and causing that increased mortality in the nation which is ever observable when a wave of cold is extensive and extreme.

I was inclined, originally, to consider that in all cases the effect of external or internal influences on the lungs was made through the action of the influencing substance on the organic nervous distribution. I thought, for example, that the effect of the amyl nitrite, and of all the nitrites so fully exemplifiable in the pulmonary structure must be due to their effect on the organic nervous fibres distributed through the structures; to the capillaries of the pulmonary artery and pulmonary veins, and to the bronchial mechanism. It was urged against my views in this respect that the distribution of sympathetic fibres was too imperfect to admit of the argument in full force, and that irritation of nervous trunks did not produce the changes in the pulmonic tissue that are observed when irritation is applied to other parts in which there is a freer distribution. To some extent there must be, I still think, some effect incident to nervous sympathetic supply, sufficient, that is to say, to account for nutritive changes of structure; but, on further consideration, I am in doubt whether direct physical impression on nervous matter is sufficient to account for all the physical demonstrations of pulmonic congestion or collapse we meet with in disease.

On such further consideration I think it right to revise my views on the subject in hand, and their bearings on the therapeutical question. The anatomical argument on the point no doubt influences the mind very much, for there is no overcoming the fact that the nervous influence beyond the changes in nutrition must be very limited in lung structure. But there is something that goes beyond this. If we reflect, it must appear to us extremely improbable that such exposed and vital structures as the lungs should, with safety, remain under the same direct control as other structures. If, for instance, the vessels of the lungs could be influenced like the vessels of the cheek, there might be a temporary congestion with every blush, or if the extreme bronchial channels were open to the impression varied with the many surrounding changes to which the body is exposed, there would certainly be under every sudden change from heat to cold a distinct mechanical variation in the process of oxidation. Again, if the pulmonic structures were directly influenced by medicinal substances there would be marked modifications of breathing which would tell the story in physical change of disarrangement in the lungs, either in their circulation or in their bronchial mechanism. I incline, therefore, now to the view that in studying the therapeutics of the lesser circulation we are bound, in a large degree, to consider acute pulmonary perversions as due, most of all, to the power exerted by the heart in driving the column of blood from the right to the left side.

In relieving the lungs, consequently, from congestion of blood we must look primarily to the action of the heart and the volume of blood with which it is charged; that is to say, in reducing congestion of the lung we must either, by increasing the tonicity of the heart cause the blood to flow over the pulmonary circuit with greater rapidity and completeness, or we must reduce the volume of the column of blood so that the lesser circulation may be subjected to practical venesection through the general circulation. This accords well with what has to be observed in practice. If there be any two remedies of real value in the treatment of congestion of the lungs those two remedies are such medicines as digitalis or strophantus, which by increasing the ventricular power, more rapidly fill and exhaust the lungs; or venesection, and the removal of oppression by the abstraction of blood from an enfeebled and overcharged right side of the heart.

